# Complement analysis reveals new biomarkers in patients with juvenile idiopathic arthritis

**DOI:** 10.1186/1546-0096-12-S1-P32

**Published:** 2014-09-17

**Authors:** Juergen Brunner, Thomas Giner, Reinhard Wuerzner

**Affiliations:** 1 Medical University Innsbruck, Innsbruck, Austria

## Introduction

The juvenile idiopathic arthritis is a well researched disease in the group of autoimmunopathies. Beside the deregulation of T-cells and cytokines also the complement system is involved in the pathogenesis of this group of diseases.

## Objectives

This prospective longitudinal study investigated the contribution of the complement system in patients with juvenile idiopathic arthritis, using practicable ELISA techniques (Wieslab® screening kit; SC5b9 soluble terminal complement complex ELISA).

## Methods

Serum and plasma of the peripheral blood and the synovial fluid were investigated for the activity of the three complement pathways - classical (CP), mannose binding lectin (MBL), and the alternative pathway (AP) and total complement activity by measuring SC5b9. Results where compared to published reference controls and 18 children without activation of inflammation as an age matched control group.

In total 57 samples of peripheral blood (PB) and 8 samples from synovial fluid (SF) from 28 children with JIA were investigated in a longitudinal observation during acute phase and remission.

## Results

The screening of complement system showed debasement of the AP (8 of 10) and CP (7 of 10) in patients during acute phase (7 of 10). The SC5b9 measurement showed a significant (p<0.002) higher amount in plasma (3,6AU/ml in median) and serum (31,4AU/ml) during acute phase compared to the control group (serum - 7,72AU/ml and plasma – 1,25AU/ml in median).

## Conclusion

In conclusion the study confirmed, that the CP and AP of the complement system are main contributors in the pathogenesis of JIA. Because of significant elevation of SC5b9 in acute phase of JIA, complement blockade with Anti-C5 may be a therapeutically option in the future.

## Disclosure of interest

None declared.

